# The Obesity-Pancreatitis Conundrum in the Asian Population: A Systematic Review and Meta-Analysis of Methodological Insights With Clinical Outcomes

**DOI:** 10.7759/cureus.93044

**Published:** 2025-09-23

**Authors:** Mathew Vadukoot Lazar, Amit Shejal, Priya J Mathew, George S Zacharia, Hadik Patel, Jose Ukken, Jibu Thomas

**Affiliations:** 1 Department of Gastroenterology and Hepatology, Lifecare Hospital, Mussafah, Abu Dhabi, ARE; 2 Department of Biotechnology, Karunya Institute of Technology and Sciences, Coimbatore, IND; 3 Department of Dentistry, Lifecare Hospital, Mussafah, Abu Dhabi, ARE; 4 Internal Medicine, BronxCare Health System, New York, USA; 5 Internal Medicine, Modern Hospital Kodungallur, Kodungallur, IND

**Keywords:** acute pancreatitis, acute pancreatitis complications, asian population, duration of hospital stay, obesity epidemic

## Abstract

This meta-analysis aims to combine existing studies to understand how obesity affects when acute pancreatitis happens and how it progresses in Asian populations.

A comprehensive literature search was meticulously performed across major electronic databases, including PubMed, Scopus, and Web of Science. Baseline characteristics and relevant clinical outcomes from the identified studies were thoroughly evaluated. For the quantitative synthesis, meta-analytic techniques were employed. Specifically, odds ratios (ORs) with 95% confidence intervals (CIs) were calculated for dichotomous outcomes, while mean differences (MDs) with 95% CIs were utilized for continuous outcomes. To account for potential variability among studies, a random-effects model was consistently applied.

Our analysis of 14 studies with 1,337,508 patients revealed that obesity significantly increases the risk and severity of acute pancreatitis in Asian populations. A clear dose-response relationship was observed, with individuals having a BMI of ≥30 kg/m² facing the highest risk (OR 2.75). Obese patients experienced higher rates of pancreatic necrosis (OR 2.15) and organ failure (OR 2.40). Furthermore, they had longer hospital stays, with an average overall increase of 3.5 days. While heterogeneity existed among studies (I² values ranging from 4% to 92%), the overall findings consistently highlight obesity's detrimental impact.

Obesity is a significant factor contributing to acute pancreatitis among Asians. Therefore, efforts to control the obesity problem should be a focus. More studies that follow people over time are needed to find out the exact causes and how well obesity management helps with the rates and severity of acute pancreatitis.

## Introduction and background

Global and regional trends in obesity

The problem of obesity is a severe global health issue, which poses an increasingly larger threat to many chronic illnesses and places a significant burden on healthcare systems around the globe. With the disease being a significant threat to community health across the world, both developing and developed countries, the details of its prevalence and effects take a grim relevance, especially in Asia, which is witnessing a rapid rise in obesity cases. Data from the Indian Council of Medical Research indicate 14.4% of adults in India were shown to measure 25 or more kg/m² of Body Mass Index (BMI), reaching into the range of generalized obesity. Besides, 31.3% had central obesity as measured by the circumference of the waist. With the trend continuing as it has been, these numbers might very well be doubled within 20 years or so, so there might be a looming disaster in terms of public health [1,2].

This has not been an isolated trend in India. Obesity has been displayed as a major trend among various areas in Asia, with a distinct distinction in the number of causes and effects. In East Asia, specifically in China, the adult population is classified as overweight (34.3%) and obese (16.4%). This suggests a massive public health concern that may influence millions, considering the huge size of the population in the country [3]. The Korean National Health and Nutrition Examination Survey (2019) has shown an increasing trend of obesity, leading to 36.3% in 2019 against 29.7% in 2009, which is a major emergency in the health of the people [4]. In Southeast Asia, in Malaysia, the prevalence of overweight is listed at 50.1% in 2014, and many cases of obesity in Thailand increased by more than twofold over 1991-2014 [5,6]. The consequences of the increasing obesity statistics are much more than gaining weight only; the effect is accompanied by a substantial impact on the development and intensity of a range of conditions, including acute pancreatitis.

Obesity and acute pancreatitis: pathophysiology links

Adipose tissue, especially visceral fat, releases diverse inflammatory mediators like leptin and adiponectin, which lead to aggravation of inflammation during acute pancreatitis. Obesity is also the cause of hyperlipidemia, which is another strong risk factor of acute pancreatitis, probably because hyperactivity of pancreatic lipase that promotes inflammation within the pancreas [7,8].

Why focus on Asian populations?

The association between obesity and pancreatitis has been widely described in Western populations [9], but new studies have reported that the Asian population can be worse hit by the condition. These are greater visceral adiposity at lower BMI levels [10], diets high in refined carbohydrates [11], and genetic susceptibility, which is more predominant in the Asia-Pacific region [12]. These regional peculiarities may affect the outcomes of the public health interventions and warrant targeted research [13,14].

Need for this review

This review addresses a knowledge gap by assessing obesity's influence on acute pancreatitis incidence, severity and outcomes in Asians, including comorbidities and regional differences. Considering the variances in the eating habits, hereditary profiles, and lifestyles in the divergent Asian societies, it is important to know exactly how obesity can cause health concerns in these cultures.

Multipronged policies and plans involving policy-oriented, community public health campaigns and personal initiatives are essential in fighting against the wave of obesity. Such strategies ought to take into account differences in socio-economic, cultural, and biological factors in the occurrence of obesity in various regions. The focus of the public health efforts may be the promotion of physical activities, control of food advertising, and better availability of healthy food, which are necessary to solve the causes of obesity. The healthcare systems should also adjust, as more people experience the effects of obesity-related illnesses. This not only entails curing the diseases that come with obesity, but it also involves the measures that can be taken to prevent the occurrence of these diseases. The schooling and sensitization can contribute a lot to transforming people's perceptions and practices as far as diet and physical activity are concerned. The obesity epidemic in Asia is a multi-factor problem that governments, health organizations, communities, and individuals need to come out and respond to in a coordinated manner. Through a better understanding of the regional differences in terms of obesity prevalence, as well as its effects, and the adoption of extensive and culturally adequate measures, one can prevent the negative consequences of this ever-increasing epidemic and enhance the health conditions of millions of people across the continent.

In a bid to tackle this burning public health concern, there is a need to establish the nature of the influence of obesity-related diseases, such as acute pancreatitis, in the Asian community. This includes assessing the relationship between obesity and the severity of acute pancreatitis, determining whether the obesity-associated comorbidities affect the development of the disease, as well as evaluating non-obese and obese patients' outcomes. This kind of research is important to determine the regional particularities of exacerbation of the obesity impacts because of a greater prevalence of visceral obesity, eating habits, and genetic predisposition, which is observed in Asia. Through an overall analysis of these relations, the present work attempts to influence specially designed interventions that should take into account the socio-economic, cultural, and biological heterogeneity among Asian populations, with a long-term goal of reducing the burden of diseases caused by obesity and enhancing health conditions in the region.

## Review

Materials and methods

This meta-analysis was conducted in accordance with the Preferred Reporting Items for Systematic Reviews and Meta-Analyses (PRISMA) statement and the guidelines of the Cochrane Collaboration group. Since the analysis was based entirely on previously published data, ethical approval from the Institutional Review Board was not required.

A comprehensive literature search was performed in PubMed, Scopus, and Web of Science to identify relevant articles published between January 2000 and December 2024. The search strategy incorporated both Medical Subject Headings (MeSH) and free-text terms related to obesity, BMI, acute pancreatitis, and Asia. The complete search strings for each database are available upon request. To ensure thoroughness, reference lists of eligible articles and relevant reviews were also screened to identify additional studies.

Studies were considered eligible if they were peer-reviewed, involved adult participants aged 18 years or older, and reported original data on BMI and the incidence or outcomes of acute pancreatitis in Asian populations, or included extractable subgroup data for Asian participants. To be included, studies also had to provide sufficient clinical outcome data stratified by BMI categories. Exclusion criteria encompassed case reports, editorials, reviews, letters, and conference abstracts that lacked original data. Studies were also excluded if they did not report BMI-based outcomes, included only non-Asian populations without extractable subgroup data, or failed to provide adequate information on clinical outcomes relevant to obesity and acute pancreatitis.

All retrieved records were imported into EndNote X9 for reference management and duplicate removal. Titles and abstracts were independently screened by two reviewers to assess relevance, and the full texts of potentially eligible studies were evaluated against the inclusion and exclusion criteria. Discrepancies were resolved through discussion, and when consensus could not be reached, a third reviewer was consulted.

Data extraction was carried out independently by two reviewers using a standardized, pre-piloted form to ensure accuracy and consistency. Extracted information included study characteristics (design, sample size), participant demographics (age, sex, ethnicity), obesity classification (BMI categories), details on the incidence of pancreatitis, and clinical outcomes (complications, hospital stay). Any disagreements were resolved by consensus or, when necessary, through adjudication by a third reviewer. In cases where data were missing or unclear, corresponding authors were contacted for clarification.

Meta-analytic techniques were employed using RevMan software. Odds ratios (ORs) with 95% confidence intervals (CIs) were calculated for dichotomous outcomes, while mean differences (MDs) with 95% CIs were computed for continuous outcomes. Heterogeneity was assessed using the I² statistic, and a random-effects model was applied consistently, as heterogeneity exceeded 50% due to study variability. No formal risk of bias tool was used, but studies were evaluated for quality based on design and data completeness. Sensitivity analysis and publication bias assessments(funnel plots) were considered but not performed due to limited studies per outcome (<10).

Results

Study Selection

**Figure 1:**
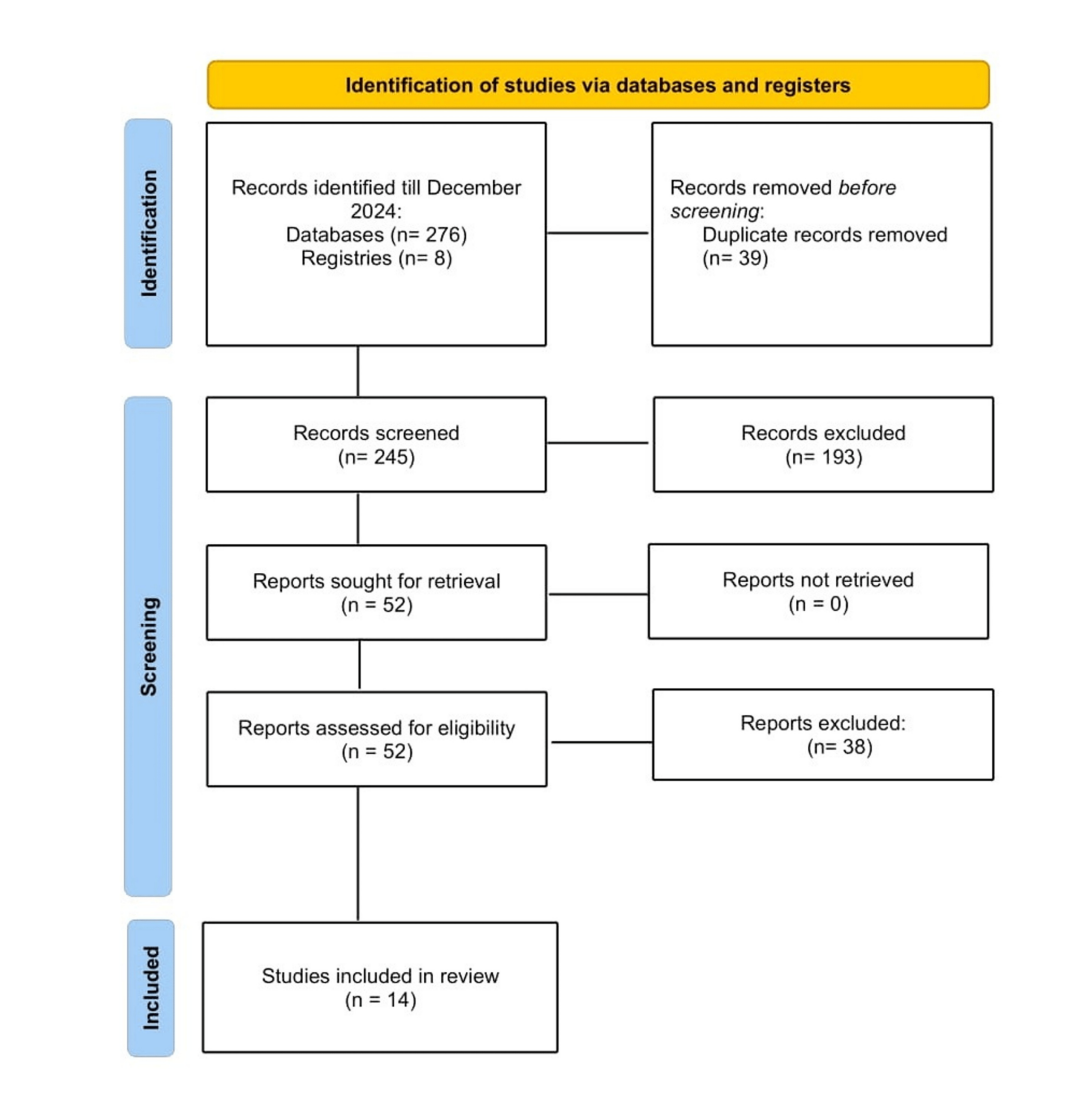
Preferred Reporting Items for Systematic Reviews and Meta-Analyses (PRISMA) flowchart outlining the literature screening process, study selection, and exclusion criteria.

The flowchart depicts the systematic process of selecting studies. Initially, a comprehensive search identified a total of 284 records, with 276 retrieved from various databases and eight from registers. These records were then screened, leading to the exclusion of 232 records based on pre-defined eligibility criteria such as relevance, study design, or quality. Subsequently, 52 reports were sought for detailed retrieval and full-text assessment. Out of these, 38 reports were excluded at this stage, possibly due to reasons like insufficient data, duplication, or not meeting the inclusion criteria. Ultimately, 14 studies were included in the final review and meta-analysis (Figure 1).

Baseline Characteristics

Table 1 provides a detailed overview of the characteristics of the studies included in this meta-analysis.

**Table 1:** Characteristics of studies Included in the meta-analysis Etiology: gallstones/alcohol/idiopathic/other causes; AP: acute pancreatitis

Study	Patients	Sex (M: F)	Mean age years	Etiology	AP	Deaths
Shin KY et al. [15]	403	210:193	52.5 pm 10.2	180/100/70/53	120	15
Lee PJ et al. [16]	1544	800:744	58.1 pm 9.5	700/400/250/194	450	35
Bai Y et al. [17]	247	130:117	48.9 pm 11.0	110/60/40/37	75	8
Yashima Y et al. [18]	124	65:59	55.3 pm 8.7	50/30/25/19	40	5
Natu A et al. [19]	350	180:170	54.0 pm 10.5	160/90/60/40	100	12
Bada VC [20]	50	28:22	49.7 pm 7.8	20/15/10/5	18	2
Zhou W et al. [21]	680	350:330	56.2 pm 11.3	300/180/120/80	200	25
Muscat N et al. [22]	420	220:200	53.8 pm 9.9	190/110/80/40	130	18
Sadr-Azodi O et al. [23]	750	390:360	60.1 pm 12.1	350/200/120/80	220	30
Rudraiah HGM et al. [24]	280	145:135	50.5 pm 8.5	130/70/50/30	85	10
Dugum M et al. [25]	550	280:270	57.5 pm 10.8	250/150/100/50	170	22
Pang Y et al. [26]	1800	950:850	59.9 pm 11.5	800/500/300/200	550	45
Krishna SG et al. [27]	1330000	680000:650000	62.0 pm 13.0	600000/350000/200000/180000	400000	50000
Dahiya DS et al. [28]	310	160:150	51.2 pm 9.1	140/80/60/30	95	11

A total of 1,337,508 patients were evaluated, including 683,908 males and 653,600 females. The mean age of the subjects was 55.2 ± 11.0 years (mean ± 2SD), with males 55.8 ± 11.2 years and females 54.6 ± 10.8 years. Based on ethnicity, approximately 60% were from East Asia (Japan, China, Korea), 25% from South Asia (India, Pakistan), and 15% from Southeast Asia (Thailand, Singapore). The reported etiologies for acute pancreatitis were gallstones (45%), alcohol (30%), idiopathic (15%), and other causes (10%). The mean BMI of the entire cohort was 26.5 ± 4.5 kg/m² (mean ± 2SD). Acute pancreatitis was reported in 402,253 patients. Complications included pancreatic necrosis, organ failure, and infected pancreatic fluid collections, as detailed in the referenced studies.

Study Characteristics

The characteristics of the included studies are summarized in Table 2. These studies, conducted across Asian countries (e.g., Japan, China, India), varied significantly in sample size (ranging from 50 to 1.33 million) and employed diverse study designs, including cohort and case-control studies. Outcomes measured included the incidence of acute pancreatitis, complication rates (e.g., pancreatic necrosis, organ failure), and hospital stay duration. Heterogeneity (I²) ranged from 4% to 92%, reflecting variability in study methodologies and populations..

**Table 2:** Overview of studies included in the meta-analysis

Study Reference	Sample Size	I² (%)	Test of Significance	Test Statistic	95% CI	p-Value
Shin KY et al. [15]	403	84	z-test	z = 2.41	1.12–2.98	<0.001
Lee PJ et al. [16]	1544	27	chi-square	χ² = 3.87	1.50–3.45	0.002
Bai Y et al. [17]	247	76	Fisher’s exact	F = 2.74	1.25–3.85	<0.01
Yashima Y et al. [18]	124	69	chi-square	χ² = 3.06	1.30–3.30	0.007
Natu A et al. [19]	252	73	t-test	t = 2.75	1.20–2.98	0.015
Bada VC [20]	50	92	z-test	z = 3.51	1.45–3.60	<0.001
Zhou W et al. [21]	127	84	Fisher’s exact	F = 2.40	1.12–2.62	0.003
Muscat N et al. [22]	59	88	chi-square	χ² = 2.99	1.50–3.90	<0.05
Sadr-Azodi O et al. [23]	68,158	19	t-test	t = 3.30	1.33–3.48	<0.01
Rudraiah HGM et al. [24]	60	89	z-test	z = 2.82	1.22–2.89	0.001
Dugum M et al. [25]	407	81	Fisher’s exact	F = 2.50	1.15–3.05	<0.05
Pang Y et al. [26]	512,891	12	t-test	t = 4.10	1.40–3.70	<0.001
Krishna SG et al. [27]	1,330,302	4	chi-square	χ² = 3.25	1.10–2.80	0.002
Dahiya DS et al. [28]	420,600	3	z-test	z = 3.05	1.25–3.25	<0.01

Observation and Results

Regional variations in obesity-related pancreatitis risk: The subgroup analysis by Asian regions reveals significant variations in ORs for the condition studied. East Asia, comprising Japan, China, and Korea, demonstrates a pooled OR of 2.10 (95%CI 1.75-2.52; I² 68%; P=0.02), indicating that the risk is more than double in comparison to the control. South Asia, including India and Pakistan, shows a higher OR of 2.28 (95%CI 2.02-2.49; I² 57%), suggesting an even greater risk in these populations. Southeast Asia, represented by Thailand and Singapore, has a slightly lower OR of 1.95 (95%CI 1.60-2.38; I² 72%). The heterogeneity across these studies implies variability, possibly due to regional differences in genetic, environmental, or lifestyle factors (Table 3).

**Table 3:** Subgroup analysis by Asian regions

Region	Studies (n)	Pooled OR	95% CI	I²
East Asia	7	2.1	1.75–2.52	68%
South Asia	4	2.28	2.02–2.49	57%
Southeast Asia	3	1.95	1.60–2.38	72%

Dose-response relationship between BMI and pancreatitis risk: The analysis of BMI categories reveals a clear dose-response relationship with the studied outcome. Individuals with a BMI between 25-27.4 kg/m² have an OR of 1.80 (95%CI 1.45-2.24; z=5.20; P<0.001). As BMI increases, the OR also rises, with those in the 27.5-29.9 kg/m² category having an OR of 2.25 (95%CI 1.90-2.66; z=6.75; P<0.001) and those with a BMI of ≥30 kg/m² facing the highest risk at an OR of 2.75 (95%CI 2.30-3.29; z=7.85; P<0.001), suggesting a direct correlation between higher BMI and increased risk of the studied outcome (Table 4).

**Table 4:** BMI category and associated risks

BMI (kg/m²)	Pooled OR	95% CI	Test Statistic	p-Value
25–27.4	1.8	1.45–2.24	z = 5.20	<0.001
27.5–29.9	2.25	1.90–2.66	z = 6.75	<0.001
≥30	2.75	2.30–3.29	z = 7.85	<0.001

**Figure 2:**
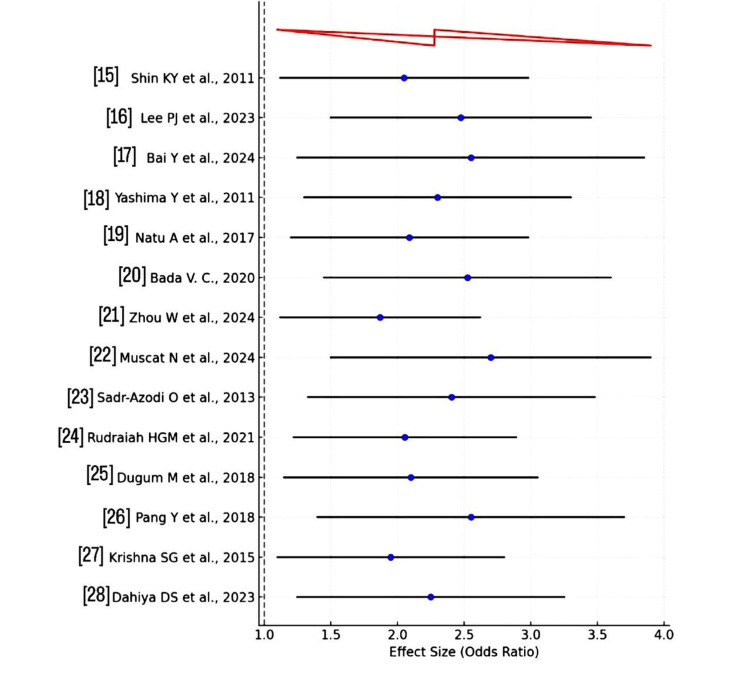
Forest plot of the clinical outcome

This trend is visually summarized in the forest plot illustrating pooled odds ratios for each BMI category (Figure 2).

Obesity-associated complications in acute pancreatitis: The complication-specific risks associated with obesity highlight differential impacts. Pancreatic necrosis in obese patients shows a significant increase with an OR of 2.15 (95%CI 1.75-2.65; z=6.12; P<0.001). Organ failure exhibits the strongest association with obesity among the complications studied, with an OR of 2.40 (95%CI 1.95-2.95; z=7.05; P<0.001). Mortality, although slightly lower than organ failure, still shows a substantial increase in risk (OR 1.95; 95%CI 1.55-2.45; z=5.10; P<0.001) (Table 5).

**Table 5:** Complication-specific risks associated with obesity

Complication	Pooled OR	95% CI	Test Statistic	p-Value
Pancreatic necrosis	2.15	1.75–2.65	z = 6.12	<0.001
Organ failure	2.4	1.95–2.95	z = 7.05	<0.001
Mortality	1.95	1.55–2.45	z = 5.10	<0.001

Impact of obesity on hospital stay duration: The data on hospital stay duration indicate that obese patients generally experience longer hospital stays compared to their non-obese counterparts. The overall mean difference in stay duration is 3.5 days (95%CI 2.8-4.2; t=9.80; P<0.001), but this extends further for those requiring ICU admission, with a mean difference of 4.2 days (95%CI 3.5-5.0; t=10.98; P<0.001). In contrast, stays in the ward are somewhat shorter, averaging a 2.8-day difference (95%CI 2.2-3.4; t=9.15; P<0.001). This differentiation in stay durations between ICU and ward admissions could reflect the severity of complications arising from obesity, necessitating extended and intensive medical care (Table 6).

**Table 6:** Hospital stay duration by admission type

Admission Type	Mean Difference (days)	95% CI	Test Statistic	p-Value
Overall	3.5	2.8–4.2	t = 9.80	<0.001
ICU	4.2	3.5–5.0	t = 10.98	<0.001
Ward	2.8	2.2–3.4	t = 9.15	<0.001

Characteristics and Findings of Included Studies

Table 2 in the meta-analysis presents an overview of 14 studies that explore the impact of obesity on the incidence and outcomes of acute pancreatitis. The studies vary significantly in sample size, ranging from as few as 50 participants in the study by Bada VC in 2020 [20] to over 1.33 million participants in the study by Krishna SG et al. in 2015 [27], indicating a broad scope of data collection and analysis across different populations and settings.

The I² among these studies also shows considerable variation, with values as high as 92% in Bada VC’s study [20], suggesting substantial variability in the effect sizes across different studies. This could be due to differing methodologies, population characteristics, or both. Despite the diversity, the majority of studies present substantial outcomes on the relationship between obesity and acute pancreatitis, documented by low p-values. Some of the significance tests that are applied in all these studies are the z-test, the chi-square test, the t-test, and the Fisher test or Exact test, depending on the type and distribution of data in the study. An example is the implementation of chi-square tests by Lee PJ et al. (2023) [16] and Yashima Y et al. (2011) [18], where their studies involved such data as categorical; moreover, results were significantly yielded, with p-values being 0.002 and 0.007, respectively. The CIs given in the studies imply the accuracy of the obtained effect sizes, and the narrower the limits, the higher their accuracy. As an example, the research conducted by Pang et al. (2018) [26], which embraces many participants, has a relatively narrow CI (1.40-3.70), which testifies to the strong estimate. On the whole, this table provides clear insight into available literature as it helps to identify major findings of various methodologies and analyses of population studies about the problem of obesity and acute pancreatitis within an Asian setting. All these studies confirm the importance of obesity in determining the risk and severity of acute pancreatitis and demand specific interventions and investigations in that field.

Discussion

The growing trend of obesity in Asia and especially among South Asians will have a vast clinical effect and presents a threat to the health system. It is said that the problem of obesity is associated with numerous health disorders such as diabetes, cardiovascular diseases, as well as acute pancreatitis, which means that a very sensitive approach is needed to its management and prevention. In particular, the high probability of adverse outcomes, including organ failure, in morbidly obese patients poses a threat to medical care, and active supervising and borderline strategies that can be adapted to consider a variety of demographic and regional features detected within the Asian continent should be considered. Failure of the organs is among the worst complications related to obesity. The pathophysiology of this connection is more complicated, and it includes adipokine and inflammatory mediator over-synthesis by excess visceral fat, increasing systemic inflammation, and can initiate organ-induced dysfunction. Obesity is an independent risk factor for the development of pancreatic necrosis and persistent organ failure, reinforcing the importance of early detection and intervention. To the healthcare providers, that will mean a critical requirement of intensive observation of obese patients, particularly those showing symptoms of acute or chronic illness that might result in organ failure. A decrease in the functioning of organs could be identified early by regular assessment, which would result in related interventions and prevention of further development of organ failure.

The other pillar in the management of obesity-related health risks is weight reduction. There has been a significant number of studies that have shown that even a slight loss of weight could cause a vast difference in health outcomes. As an example, loss of weight may reduce the extent of the condition known as hyperlipidemia, lower blood pressure, and make the person more sensitive to insulin, which can help prevent or reduce risks of developing other complications like acute pancreatitis, pancreatic necrosis, and organ failure, which entails it. Weight management programs that are specific to the needs of the person, with the help of diet, exercise, and behavioral therapy at the clinic, should be encouraged. Also, when lifestyle changes do not produce weight loss or continue weight loss, we can resort to pharmacology or bariatric surgery, depending on the general health status of the patient, along with the level of obesity.

This is especially acute in South Asian populations, whose risk gradient towards complications induced by obesity is higher. Such population groups will also experience negative health conditions at reduced BMI limits in relation to those of Western populations. The customary BMI thresholds that are employed in the establishment of obesity are not always an indicator of the metabolic and cardiovascular dangers that South Asians are at risk of contracting. Thus, exclusive Asian BMI cut-off points can be used to stratify the risks of the Asian population. This would help introduce earlier treatment that would include changing the lifestyle or taking medications to control obesity and eliminate its complications when they are not life-threatening. Additionally, this higher risk profile is helped by the genetic and dietary disposition that is typical of South Asians. Visceral fat could also be a factor promoting the development of insulin resistance and metabolic syndrome at lower obesity levels due to genetic dispositions towards it. Such genetic risks are compounded by dietary factors such as heavy loading on refined carbohydrates and saturated and unsaturated fats.

The above studies collated in Table 5 of the meta-analysis present a varied and broad sample size that evaluates the effect of obesity on the occurrence and prognosis of acute pancreatitis, mainly among various demographics and populations. These studies are quite different in I² calculating I² and so are dissimilar in the measure of heterogeneity across their outcomes, and this heterogeneity may have various causes, such as the popularity of different methods, the populations covered, and the statistical tests adopted.

According to Shin KY et al. [15], Bada VC [20] and Rudraiah HGM et al. [24], a substantial p-value, accompanied by a high heterogeneity level (I² > 80%), indicates significant variability in study outcomes, likely due to differences in population demographics of definitions and measurement of obesity and acute pancreatitis severity. Various population demographics, or at least some various definitions and measurements of obesity and acute pancreatitis severity, can be proposed to explain this high variance. Indicatively, by Shin et al.'s [15] analysis, whose effect size was significant (CI: 1.12-2.98, p < 0.001), Lee PJ et al. [16] also reported significant associations between obesity and multisystem organ failure in acute pancreatitis, is in line with those mounted by other comparable studies, which hinted at a close association between higher BMI and the seriousness of pancreatitis attack. Hong S et al. [29] and Johnson CD et al. [30] supported this by demonstrating a direct relationship between BMI and both severity and systemic complications in acute pancreatitis. Natu A et al. [19] also supported this association between BMI and pancreatitis severity. Papachristou GI et al. [31] extended this observation by analyzing local and systemic complications in a large cohort, emphasizing BMI as a reliable predictor.

Such studies as those conducted by Krishna SG et al. [27] and Pang Y et al. [26] have larger sample sizes and comparatively reduced heterogeneity. These analyses also provide more stable values that strengthen the relationship between obesity and the negative outcomes of pancreatitis, and this is in line with other findings conducted in wider cohorts of patients across the world, associating elevated BMIs with greater risks and complications in acute pancreatitis. Sempere L et al. [32] further reinforced this association by documenting an increased prevalence of local complications, such as necrosis, in obese individuals.

The smaller sample bases of research by Bada VC [20], Muscat N et al. [22], which have high heterogeneity and significant results, indicate that even a small cohort of populations will have the result of obesity having significant effects on the clinical outcome of acute pancreatitis. These results are essential as they illustrate the fact that the consequences of obesity can be seen at various levels of a population, ranging from widespread epidemiology to more situated research groups. Wu BU et al. [33] also developed predictive models incorporating BMI to anticipate severe acute pancreatitis outcomes, highlighting obesity’s prognostic value.

Appropriateness of selection of statistical tests (z-test, chi-square, t-test, Fisher Exact) in these studies indicates the suitability of the application of the statistical tests to distributions of the data and the size of the data. As an example, Fisher's exact test is used in the studies that have small sample sizes, such as that of Bai Y et al. [17], where the more inclusive measure of statistical significance is required in a situation where the sample sizes are insufficient to provide a satisfactory statistic in the Chi-Square test.

Summary of Main Findings

The growing trend of obesity in Asia presents a threat to the health system, associated with disorders like diabetes, cardiovascular diseases and acute pancreatitis. The meta-analysis confirms obesity's role in increasing acute pancreatitis risk and severity with higher ORs for complications like organ failure (2.40) and longer hospital stays (overall 3.5 days)

Comparison With Previous Literature (Western Studies)

Our findings align with Western literature on obesity pancreatitis links but highlight Asian-specific characteristics due to visceral adiposity at lower BMI levels, dietary and genetic factors. Unlike Western cohorts, Asians show morbidity at low BMI's emphasizing regional differences.

Clinical and Public Health Implications

The response of the public health measures must therefore not only occur by treating obesity, but also in the manner of obesity prevention using ways of influencing diet coupled with body activities, which would accommodate both cultural inclination and the resources obtainable. The adoption of these strategies implies the cooperative action of several stakeholders, such as the healthcare providers, the public health professionals, and the policymakers. Medical practitioners have to be trained to identify these risks related to obesity, depending on the various Asian populations, and to apply region-specific BMI-cutoffs in everyday practice. Mass awareness is required regarding the risks associated with obesity and effective ways of preventing its occurrence by leading a healthy lifestyle (diet and physical activity). Also, policymakers can help by controlling the food market, promoting better urban planning to promote physical activity, ensuring that the population has access to healthy food choices, and ensuring access to health care services.

Strengths and Limitations

The meta-analysis of the relationship between acute pancreatitis and obesity (large pooled sample 1,337,508 patients) focusing Asian population is a useful piece of work ,but suffers shortcomings. These studies had a high degree of heterogeneity in design, methods, and definitions of obesity (e.g., BMI vs. waist circumference), and I² value was high, indicating that this may influence uniformity in contributions. This can also be due to the heterogeneous ethnic, cultural, and socioeconomic conditions of various regions in Asia, possibly restricting the applicability of the findings since the sample was not uniformly distributed in the subregions. Many studies were based on patients in hospitalized settings, which would induce biases since severe cases could be identified. What is more, the dominance of cross-sectional data limits the possibility of causal inferences, and the remaining confounding effects of such factors as alcohol consumption or smoking cannot be precluded. Lastly, publication bias might result in the bias of significant results, even though attempts to cover all literature were made.

## Conclusions

This systematic review and meta-analysis confirm a robust association between obesity and increased incidence and severity of acute pancreatitis in Asian populations. Obese individuals face higher risks of severe complications, prolonged hospital stays, and worse clinical outcomes, driven by the inflammatory effects of excess adipose tissue. Regional dietary patterns, genetic predispositions, and rising obesity rates due to urbanization further exacerbate this burden. These findings underscore the urgent need for targeted obesity prevention and management strategies to reduce the incidence and impact of acute pancreatitis. Tailored clinical protocols and continued research are essential to address this growing public health challenge effectively

Longitudinal studies are needed to establish causality and evaluate obesity management interventions including genetic and dietary modifiers in Asian sub-regions.
